# Supplementary figures and images for: Novel monoclonal antibodies for immunodetection of AmpC β-lactamases
Source: PeerJ. 2025 Oct 2;13:e20036. doi: 10.7717/peerj.20036 (PMC12497404; doi:10.7717/peerj.20036)

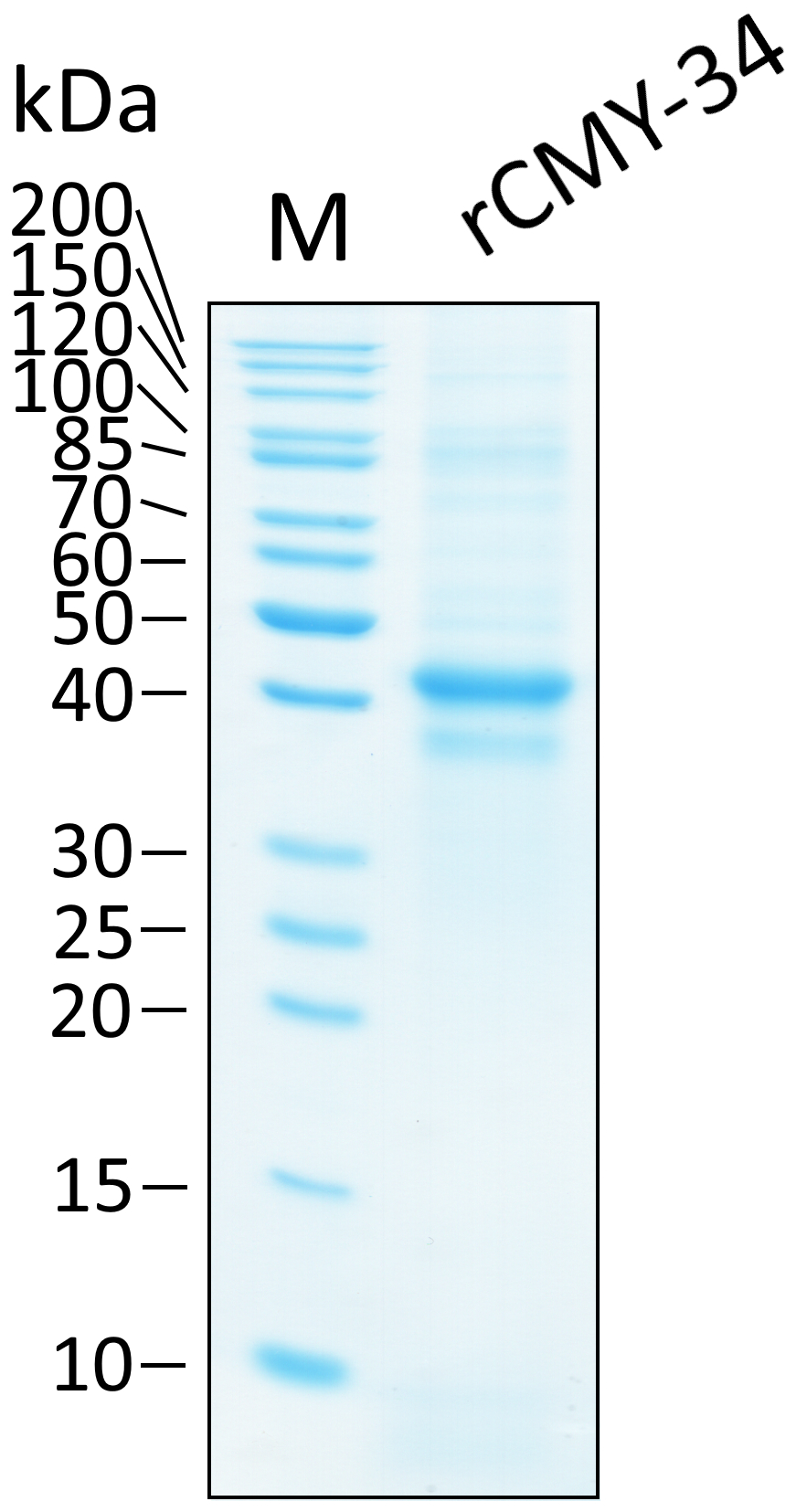

Supplement: Supplemental Information 1 — M – PageRuler™ Unstained Protein Ladder (Thermo Scientific, 26614). [file peerj-13-20036-s001.png]

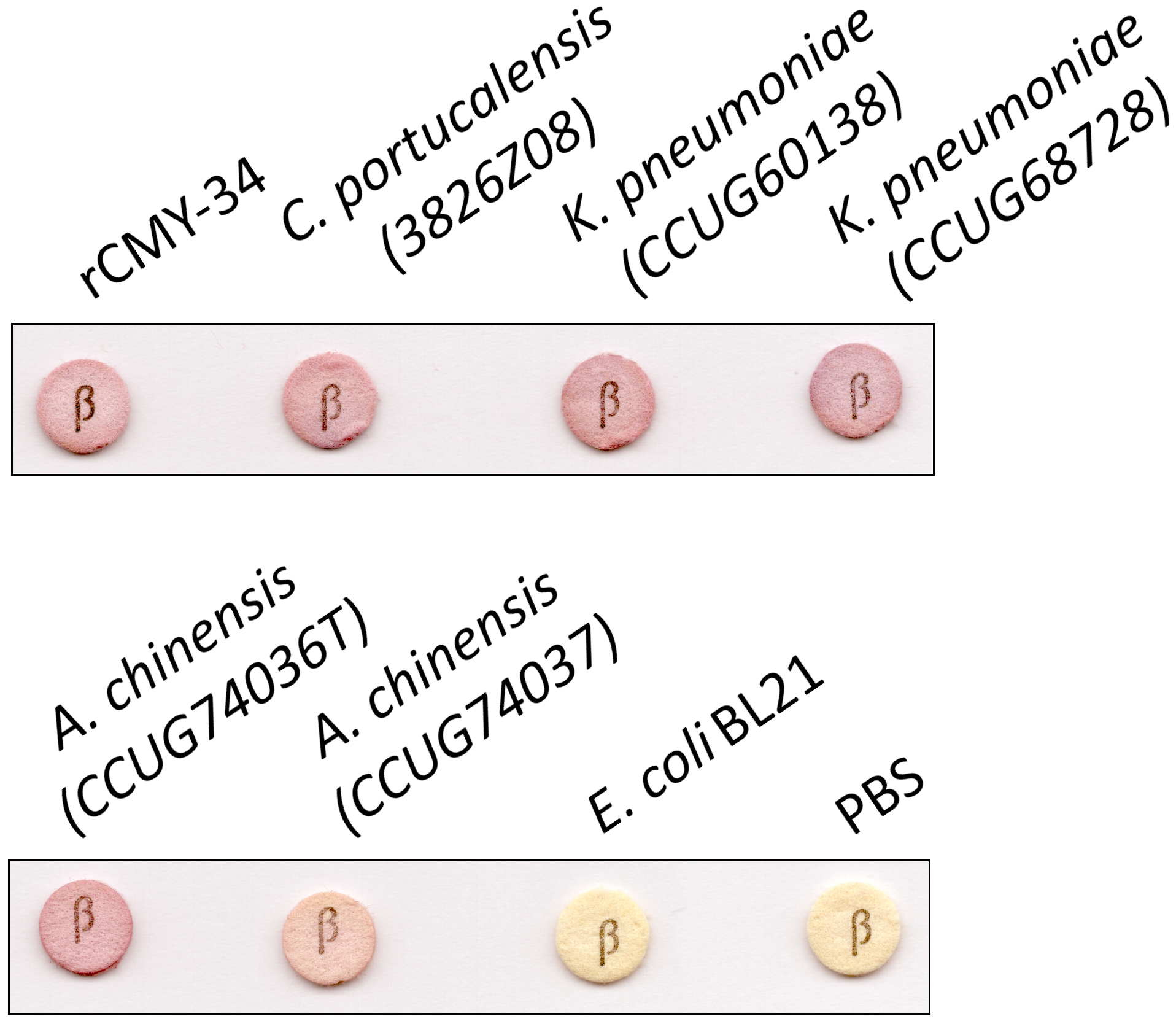

Supplement: Supplemental Information 2 — Purified rCMY-34 and prepared lysates of C. portucalensis (3826Z08, CMY-34 producer), K. pneumoniae (CCUG60138 and CCUG68728, CMY-4 and NDM-1 producers) and A. chinensis (CCUG74036T and CCUG74037, NDM-1 producers) isolates were analyzed. E. coli BL21 lysate and protein dilution buffer (PBS) were tested as negative control. Red color indicates an enzymatic activity of β-lactamases (nitrocefin hydrolysis) and yellow color indicates no enzymatic activity. [file peerj-13-20036-s002.png]

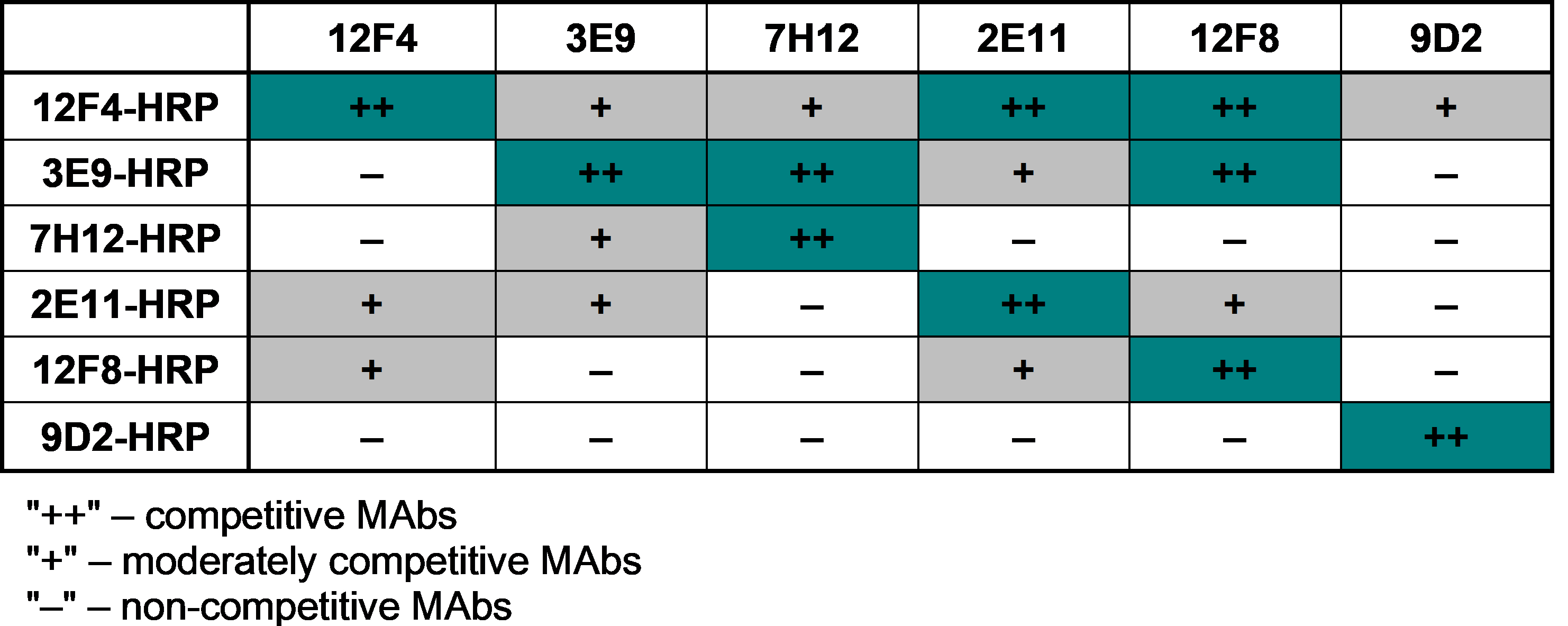

Supplement: Supplemental Information 3 — Graph demonstrates the level of MAb competition when pairs of MAbs were tested with rCMY-34. The pair is composed of HRP-conjugated MAb and unconjugated MAb. [file peerj-13-20036-s003.png]

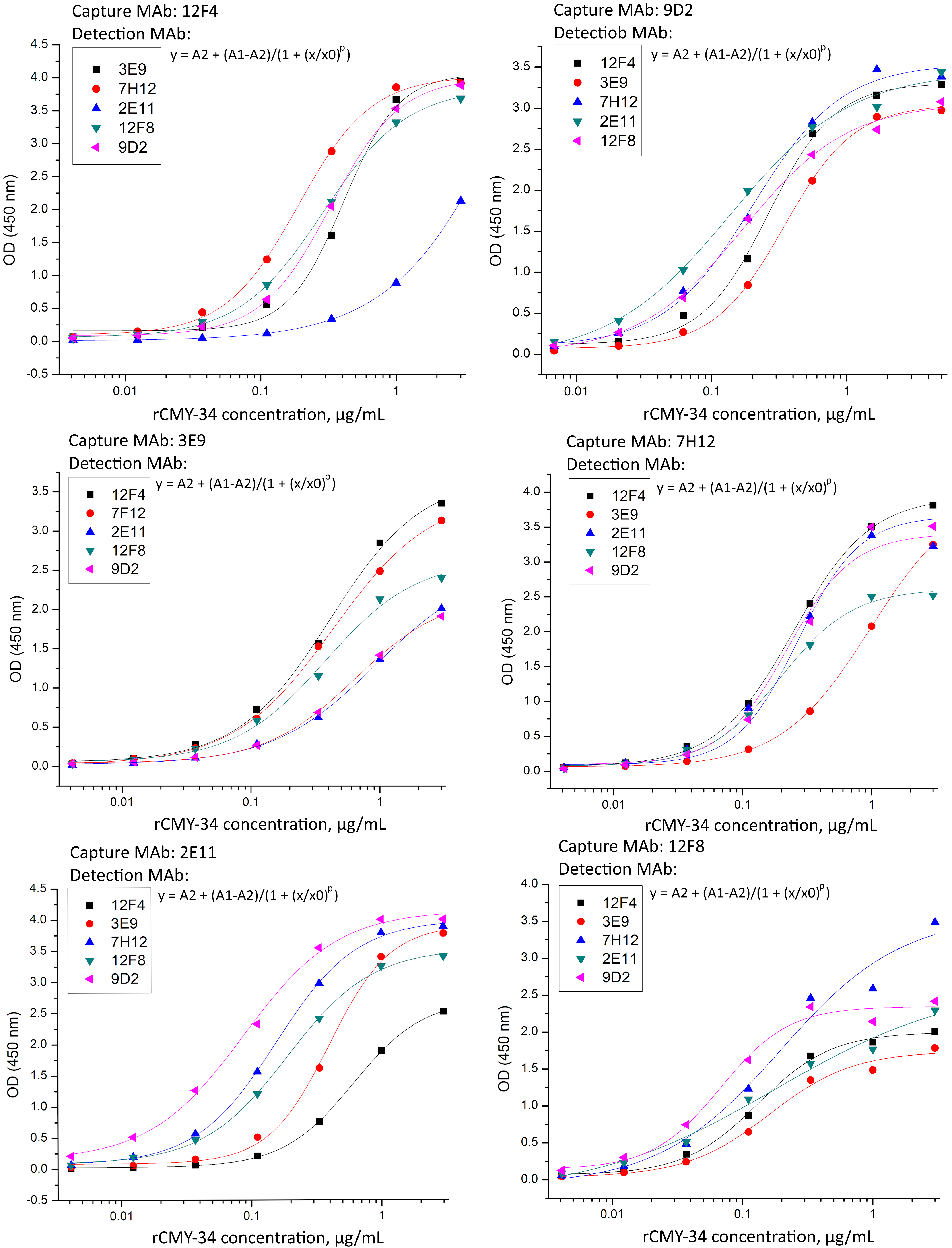

Supplement: Supplemental Information 4 — rCMY-34 was diluted in the concentration range of 3·10 1–4·10 -3 μg/mL. [file peerj-13-20036-s004.png]

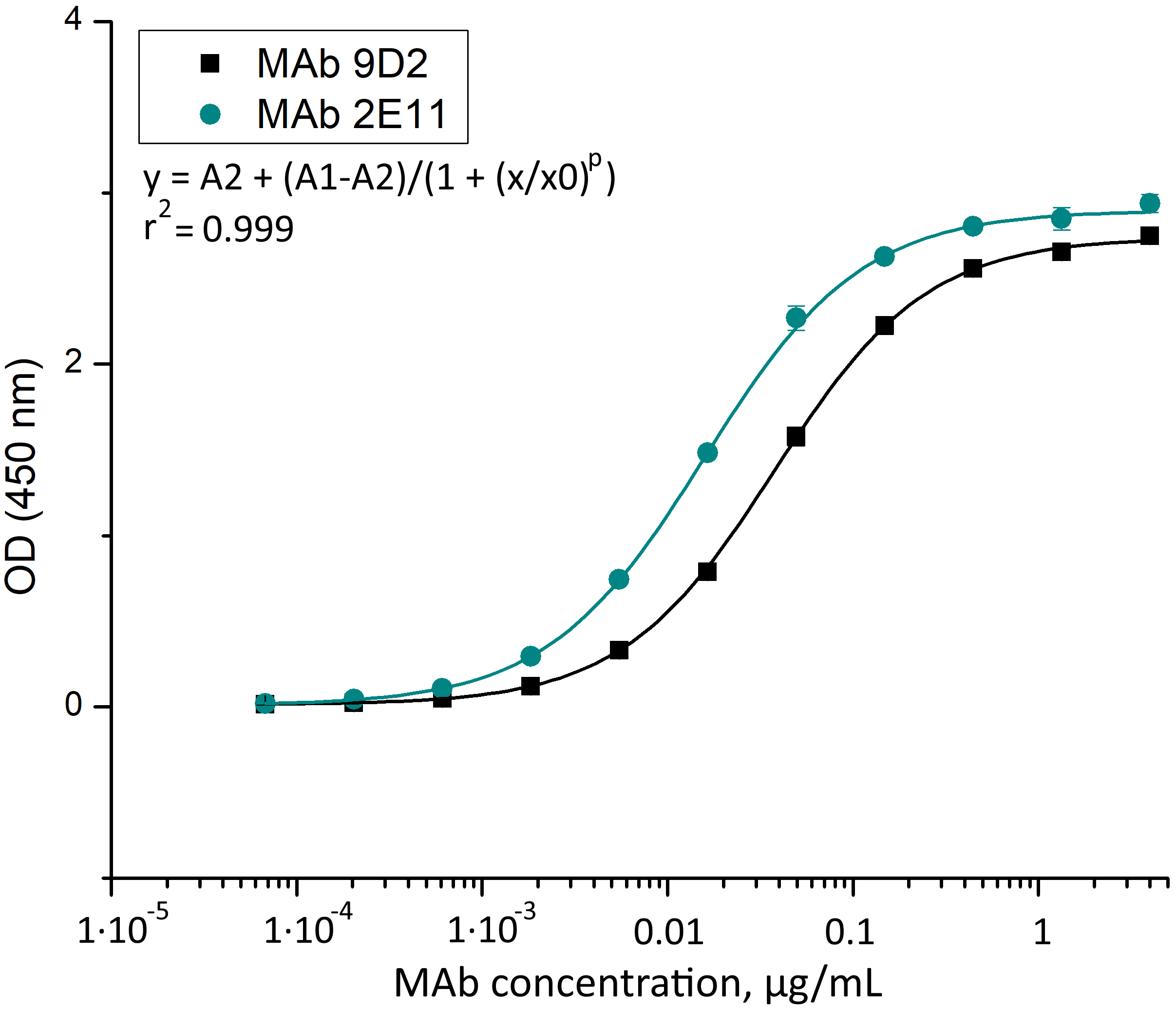

Supplement: Supplemental Information 5 — MAbs were diluted in the concentration range of 4·10 1–6.7·10 -5 μg/mL, n = 3, mean ± SD. [file peerj-13-20036-s005.png]

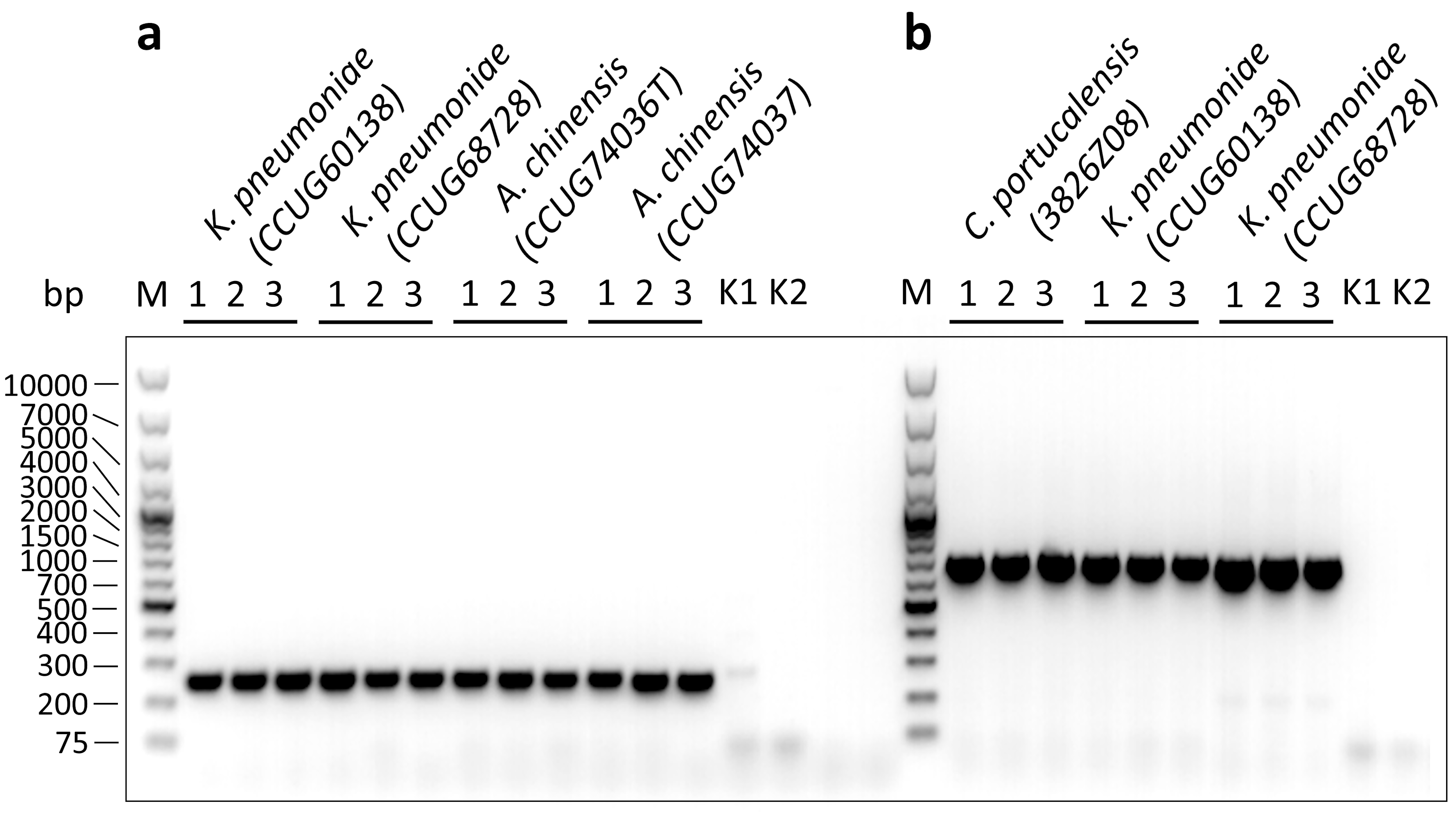

Supplement: Supplemental Information 6 — (a) Testing with blaNDM specific primers (n = 3). (b) Testing with blaCMY specific primers (n = 3). The DNA samples of characterized bacterial isolates (C. portucalensis, 3826Z08, CMY-34 producer; K. pneumoniae, CCUG60138 and CCUG68728, NDM-1 producers; A. chinensis, CCUG74036T and CCUG74037, NDM-1 producers) were tested. K1 – negative control, when tested E. coli BL21 DNA, K2 – no template control. PCR products were fractionated in 1.2% agarose gel. M – O’GeneRuler 1 kb Plus DNA Ladder (Thermo Scientific, SM1331). [file peerj-13-20036-s006.png]

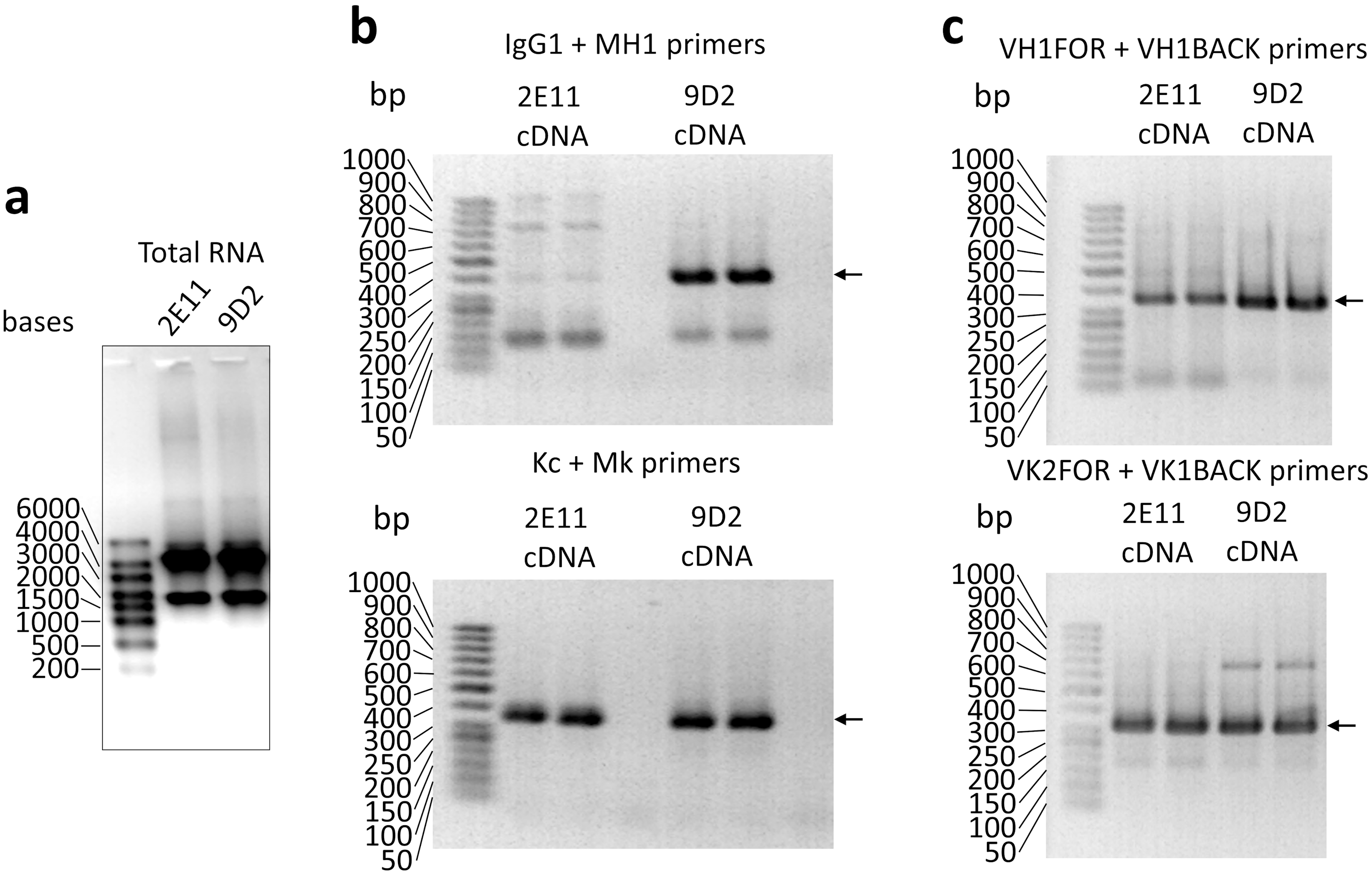

Supplement: Supplemental Information 7 — (a) Evaluation of isolated hybridoma total RNA by RNA electrophoresis. RNA samples were fractionated in 1 % agarose gel. M—RiboRuler High Range RNA Ladder (Thermo Scientific, SM1821). (b, c) Examples of amplified VH and VL coding sequences ( marked with arrows) using modified IgG1, MH1 and Kc, Mk pairs of primers (b), VH1FOR, VH1BACK and VK2FOR, VK1BACK pairs of primers (c). PCR products were fractionated in 2% agarose gel. M—GeneRuler 50 bp DNA Ladder (Thermo Scientific, SM0373). [file peerj-13-20036-s007.png]

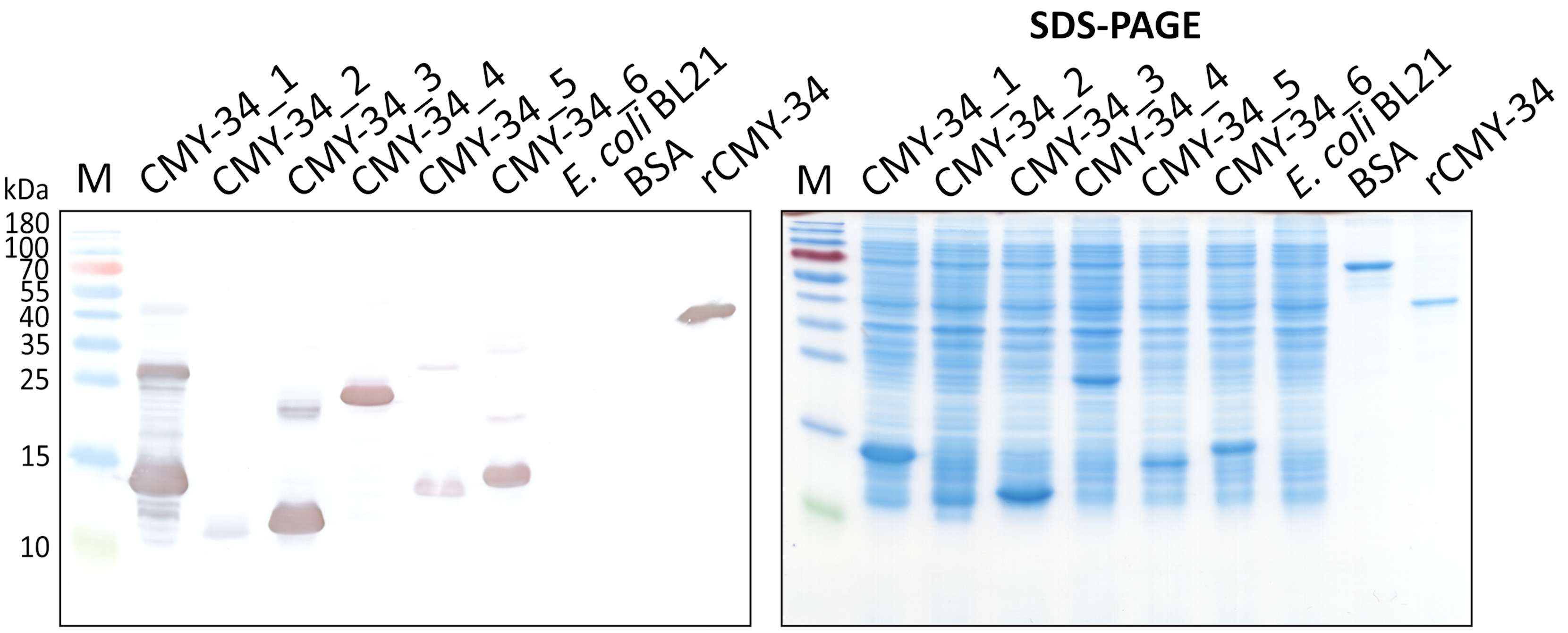

Supplement: Supplemental Information 8 — E. coli lysates after synthesis induction of CMY-34 fragments (CMY-34_1–CMY-34_6) were tested with in house-generated His-tag-specific MAb 6C2. E. coli BL21 lysate and bovine serum albumin (BSA) were used as negative controls. M—PageRuler Prestained Protein Ladder (Thermo Scientific, 26616). [file peerj-13-20036-s008.png]

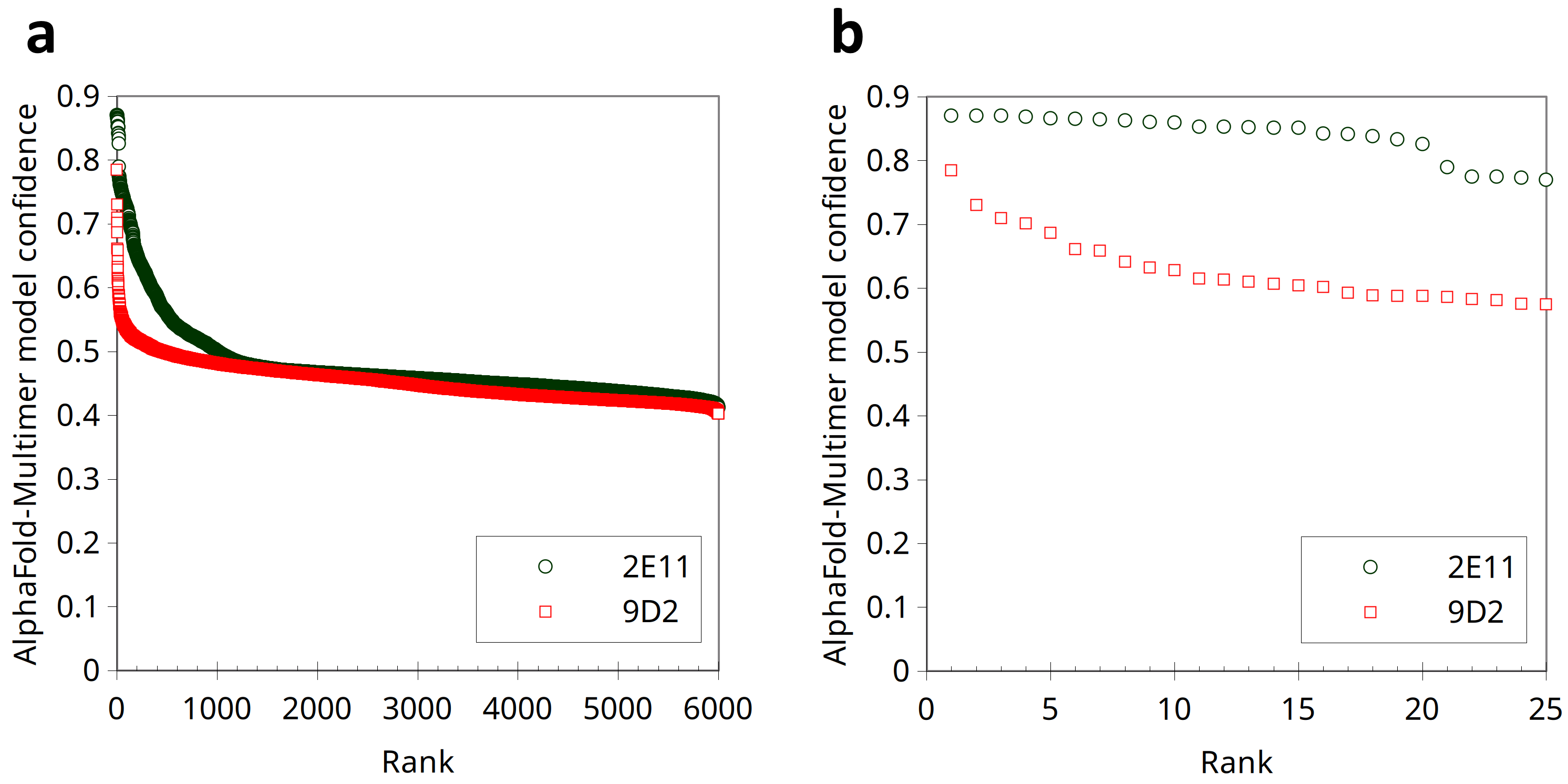

Supplement: Supplemental Information 9 — (a) Confidence scores of generated 6000 models. (b) Confidence scores of the 25 most reliable models. [file peerj-13-20036-s009.png]

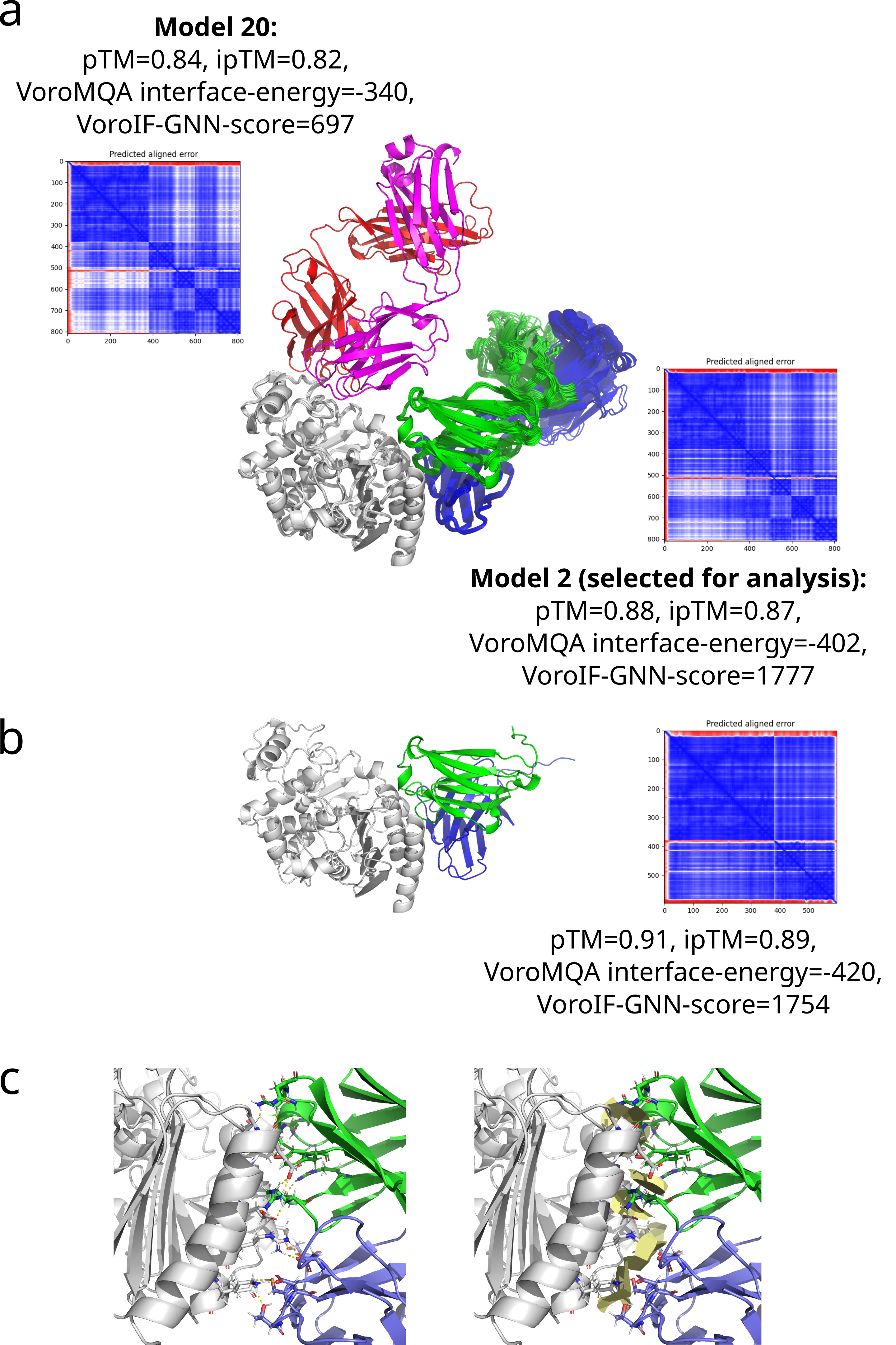

Supplement: Supplemental Information 10 — (a) 20 most reliable models that were selected according to AlphaFold accuracy self-estimates and their accuracy measures. CMY-34 is colored gray, Fab fragments in selected 19 of 20 most reliable models are colored green (heavy chain) and blue (light chain), Fab fragment in the remaining 20 th model is colored red (heavy chain) and magenta (light chain). (b) Top ranked AFsample model of the CMY-34-MAb 2E11 VH and VL interaction. (c) Hydrogen bonds at the predicted CMY-34-MAb 2E11 interface. Picture on the left demonstrates the distance-based visualization, picture on the right shows the contact areas. [file peerj-13-20036-s010.png]

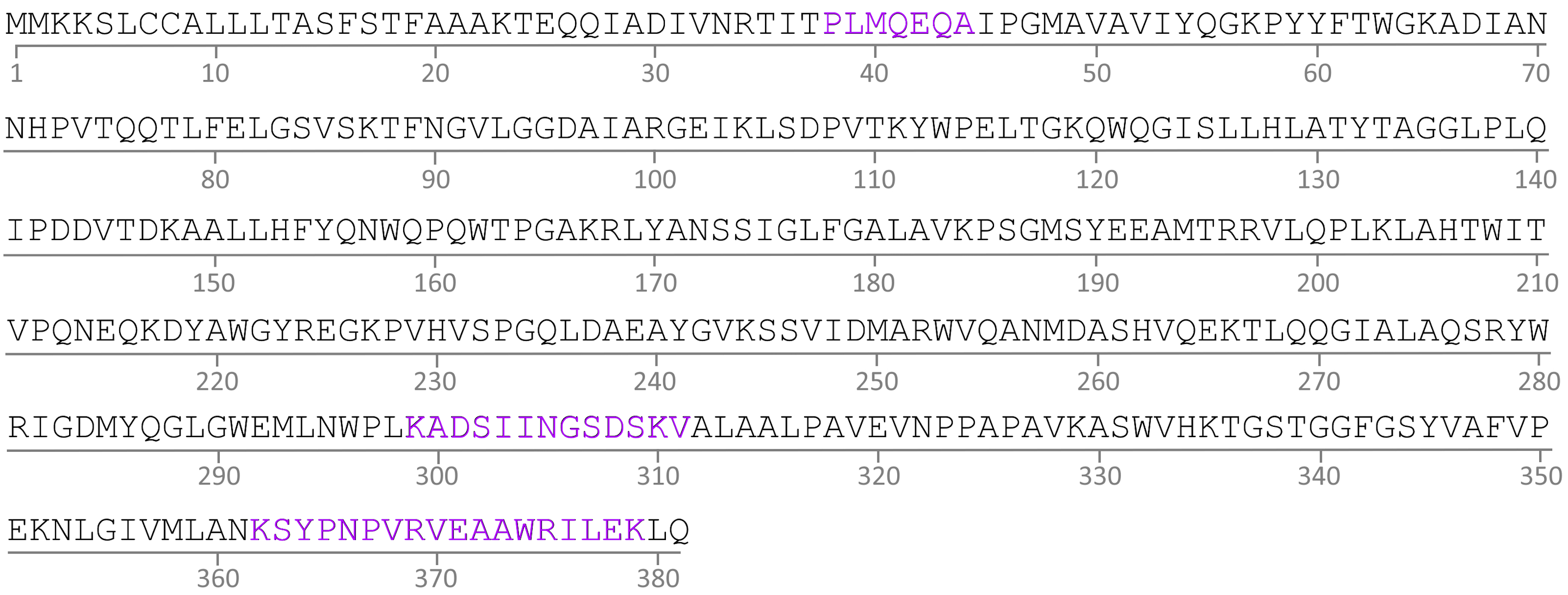

Supplement: Supplemental Information 11 — MAb 2E11 epitope is composed of three segments located at 38–44, 299–311 and 362–379 aa of CMY-34 (marked in violet). [file peerj-13-20036-s011.png]

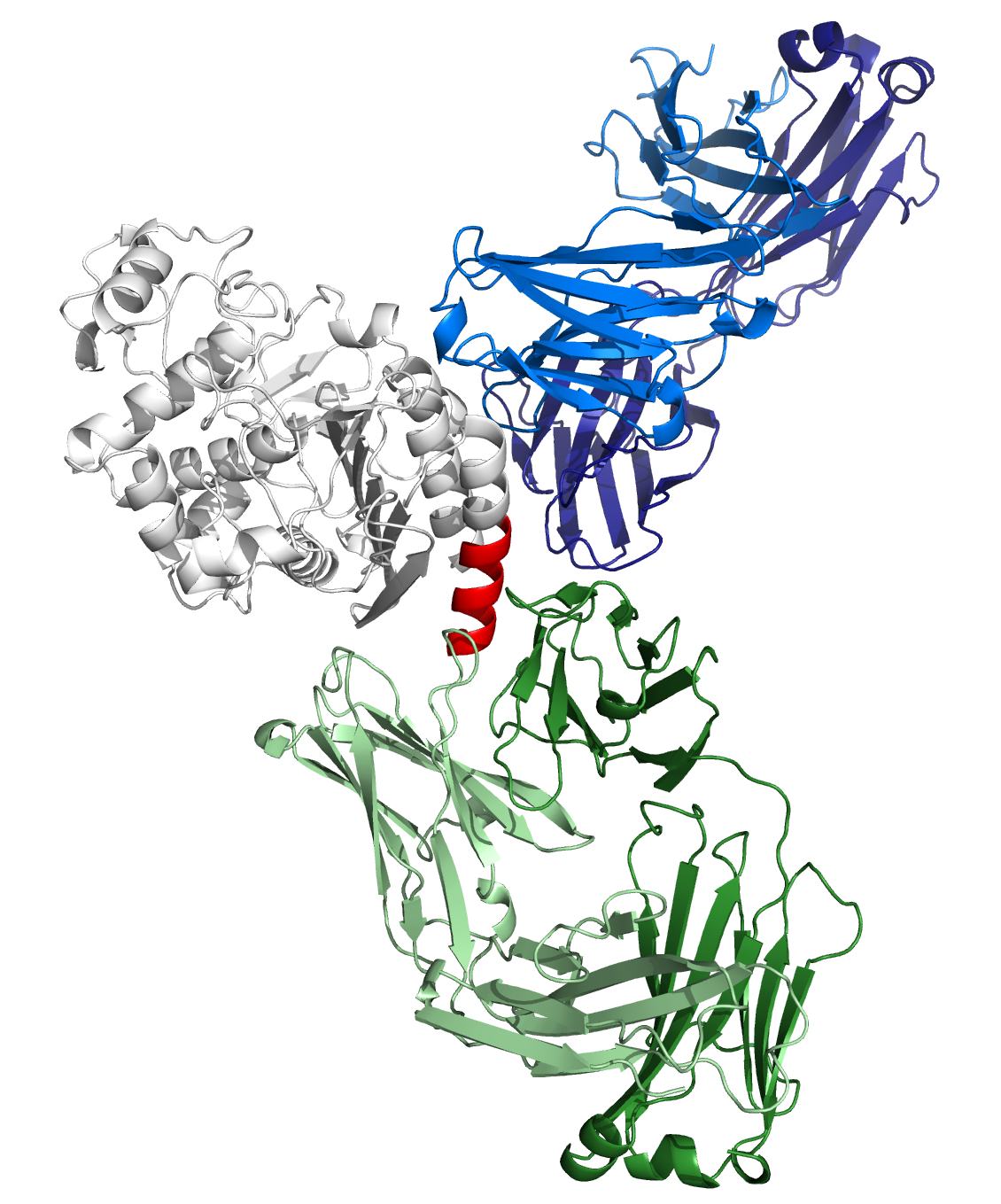

Supplement: Supplemental Information 12 — According to computational structure prediction, MAbs 2E11 (blue) and 9D2 (green) bind to different epitopes of CMY-34 (grey). Experimentally determined epitope of the MAb 9D2 is colored red. [file peerj-13-20036-s012.png]
