# Supplementary material for: Novel monoclonal antibodies for immunodetection of AmpC β-lactamases
Source: PeerJ. 2025 Oct 2;13:e20036. doi: 10.7717/peerj.20036 (PMC12497404; doi:10.7717/peerj.20036)
Supplement: Supplemental Information 13 [file peerj-13-20036-s013.docx]

| **No.** | **MAb clone** | **Isotype** | **K_d_, nM^a^** |
| --- | --- | --- | --- |
| 1. | 9D2 | IgG1 κ | 0.24 ± 0.006 |
| 2. | 2E11 | IgG2a κ | 0.10 ± 0.002 |
| 3. | 1A2 | IgG1 κ | 0.29 ± 0.023 |
| 4. | 7H12 | IgG2a κ | 0.05 ± 0.004 |
| 5. | 8G6 | IgG1 κ | 0.26 ± 0.013 |
| 6. | 7F1 | IgG1 κ | 0.10 ± 0.008 |
| 7. | 12F4 | IgG2a κ | 0.24 ± 0.011 |
| 8. | 12E5 | IgG1 κ | 0.13 ± 0.007 |
| 9. | 7D10 | IgG2a κ | 2.50 ± 0.137 |
| 10. | 3A2 | IgG1 κ | 0.10 ± 0.002 |
| 11. | 14C7 | IgG1 κ | 0.12 ± 0.006 |
| 12. | 13A8 | IgG1 κ | 0.06 ± 0.001 |
| 13. | 12F8 | IgG1 κ | 0.08 ± 0.004 |
| 14. | 3E9 | IgG1 κ | 0.18 ± 0.009 |

^a^The values of apparent dissociation constant (K_d_) were determined by indirect ELISA when rCMY-34 was tested (n = 3, K_d_ ± SEM).
