# Supplementary material for: Novel monoclonal antibodies for immunodetection of AmpC β-lactamases
Source: PeerJ. 2025 Oct 2;13:e20036. doi: 10.7717/peerj.20036 (PMC12497404; doi:10.7717/peerj.20036)
Supplement: Supplemental Information 14 — Positions of mismatched aa are marked in yellow. [file peerj-13-20036-s014.docx]

| **CMY**  **β-lactamases** | **Alignment of 299–311 aa region of CMY-34** | **GenBank no.** |
| --- | --- | --- |
| CMY-34 | 299 KADSIINGSDSKV 311 | ABN51006.1 |
| CMY-20, CMY-28,  CMY-140, CMY-146 | 299 KADSIINGSDNKV 311 | AAX58682.2, ABQ51091.1, ANJ78051.1, APD79117.2 |
| CMY-133 | 299 KADSIINGNDSKV 311 | AKZ20821.1 |
| CMY-56 | 299 KTDSIINGSDSKV 311 | ADT91162.1 |
| CMY-70, CMY-74,  CMY-82, CMY-83,  CMY-93, CMY-100, CMY-101, CMY-125, CMY-137, CMY-179, CMY-182 | 299 KADSIISGSDSKV 311 | KKC62625.1, AFU25632.1, AHL39324.1, AFU25638.1, AHM76768.1, AHA80101.1, EOQ33708.1, AKO62862.1, WP_053390271.1, MBC6501781.1, AUV24482.1 |
| CMY-157 | 299 KADTIINGSDSKI 311 | ASW32315.1 |
| CMY-18 | 299 KADSIINGNGSDSKV 313 | AAU95778.1 |
