# Supplementary material for: Novel monoclonal antibodies for immunodetection of AmpC β-lactamases
Source: PeerJ. 2025 Oct 2;13:e20036. doi: 10.7717/peerj.20036 (PMC12497404; doi:10.7717/peerj.20036)
Supplement: Supplemental Information 15 — Positions of mismatched aa are marked in yellow. [file peerj-13-20036-s015.docx]

| **CMY**  **β-lactamases** | **Alignment of 362–379 aa region of**  **CMY-34** | **GenBank no.** |
| --- | --- | --- |
| CMY-34 | 362 KSYPNPVRVEAAWRILEK 379 | ABN51006.1 |
| CMY-12, CMY-38 | 362 KNYPNPVRVEAAWRILEK 379 | CAA76382.1, CAP60699.1 |
| CMY-37 | 362 KSYPNPIRVEAAWRILEK 379 | BAF36388.1 |
| CMY-161 | 362 KSYPNPVRFEAAWRILEK 379 | AVP74338.1 |
| CMY-22 | 362 KSYPNPVRVEAYWRILEK 379 | ABB72431.1 |
| CMY-108 | 362 KSYPNPVRVEAAWHILEK 379 | AGZ20169.1 |
| CMY-174 | 362 KSYPNPVRVEAARRILEK 379 | QQL36767.1 |
| CMY-96, CMY-155 | 362 KSYPNLVRVEAAWRILEK 379 | AFZ85212.1, ALF62843.1 |
| CMY-185 | 362 KSYPYPVRVEAAWRILEK 379 | WCB91330.1 |
| CMY-172, CMY-178 | 359 KSYPIPVRVEAAWRILEK 376 | MTZ79300.1 |
| CMY-41, CMY-47,  CMY-48, CMY-51,  CMY-53, CMY-56,  CMY-66, CMY-72,  CMY-78, CMY-81,  CMY-84, CMY-87,  CMY-89, CMY-90,  CMY-97, CMY-103, CMY-109, CMY-110, CMY-112, CMY-113, CMY-114, CMY-115, CMY-117, CMY-118, CMY-128, CMY-135, CMY-150, CMY-151, CMY-152, CMY-159, CMY-168, CMY-169, CMY-170, CMY-179, CMY-180, CMY-181, | 362 KSYPNPARVEAAWRILEK 379 | BAG14343.1, ADH82410.1,  ADP02979.1, AFK73431.1,  ADQ38362.1, ADT91162.1,  AEZ49849.1, AAK32688.1,  AFK73443.1, AFK73452.1,  AFK73455.1, BAL63057.1,  CCK86742.1, CCK86743.1,  AFZ85213.1, AHA80104.1,  CAG34070.1, BAO05497.1,  AIT76090.1, AIT76089.1,  AIT76099.1, AIT76092.1,  AIT76097.1, AIT76091.1,  AKO62865.1, AKP17985.1,  ARQ85811.1, ARE65229.1,  ARM19732.1, ATD84842.1  QFR38193.1, QFR38194.1,  QFR38195.1, MBC6501781.1,  AYL78128.1, EGT0516627.1 |
| CMY-67 | 362 KSYPNPARVEVAWRILEK 379 | AFK08541.1 |
| CMY-104, CMY-157 | 362 KSYPNPARVDAAWRILEK 379 | AGR82311.1, ASW32315.1 |
| CMY-86 | 362 KSYPNPVRVEVAWHILEK 379 | AHL39327.1 |
| CMY-166 | 362 KNYPIPVRVEAAWRILEK 379 | QDM39447.1 |
